# The hazard of carbapenemase (OXA-181)-producing *Escherichia coli* spreading in pig and veal calf holdings in Italy in the genomics era: Risk of spill over and spill back between humans and animals

**DOI:** 10.3389/fmicb.2022.1016895

**Published:** 2022-11-17

**Authors:** Virginia Carfora, Elena Lavinia Diaconu, Angela Ianzano, Paola Di Matteo, Roberta Amoruso, Elena Dell'Aira, Luigi Sorbara, Francesco Bottoni, Flavia Guarneri, Laura Campana, Alessia Franco, Patricia Alba, Antonio Battisti

**Affiliations:** ^1^Department of General Diagnostics, National Reference Laboratory for Antimicrobial Resistance, Istituto Zooprofilattico Sperimentale del Lazio e Della Toscana “M. Aleandri”, Rome, Italy; ^2^Sede Territoriale di Brescia, Laboratorio Diagnostica Generale, Istituto Zooprofilattico Sperimentale Della Lombardia e Dell’Emilia-Romagna “Bruno Ubertini”, Brescia, Italy; ^3^Agenzia Tutela Della Salute, Brescia, Italy

**Keywords:** carbapenem-resistant Enterobacterales, OXA-48-like carbapenemases, OXA-181 carbapenemases, long-read sequencing, Whole Genome Sequencing, plasmids, pig, bovine

## Abstract

Carbapenemase-producing Enterobacterales (CPE) are considered a major public health issue. In the frame of the EU Harmonized AMR Monitoring program conducted in Italy in 2021, 21 epidemiological units of fattening pigs (6.98%; 95% CI 4.37–10.47%; 21/301) and four epidemiological units of bovines <12 months (1.29%; 95% CI 0.35–3.27%, 4/310) resulted positive to OXA-48-like-producing *E. coli* (*n* = 24 OXA-181, *n* = 1 OXA-48). Whole Genome Sequencing (WGS) for in-depth characterization, genomics and cluster analysis of OXA-181-(and one OXA-48) producing *E. coli* isolated, was performed. Tracing-back activities at: (a) the fattening holding of origin of one positive slaughter batch, (b) the breeding holding, and (c) one epidemiologically related dairy cattle holding, allowed detection of OXA-48-like-producing *E. coli* in different units and comparison of further human isolates from fecal samples of farm workers. The OXA-181-producing isolates were multidrug resistant (MDR), belonged to different Sequence Types (STs), harbored the IncX and IncF plasmid replicons and multiple virulence genes. Bioinformatics analysis of combined Oxford Nanopore Technologies (ONT) long reads and Illumina short reads identified *bla*_OXA-181_ as part of a transposon in IncX1, IncX3, and IncFII fully resolved plasmids from 16 selected *E. coli,* mostly belonging to ST5229, isolated during the survey at slaughter and tracing-back activities. Although human source could be the most likely cause for the introduction of the *bla*_OXA-181_-carrying IncX1 plasmid in the breeding holding, concerns arise from carbapenemase OXA-48-like-producing *E. coli* spreading in 2021 in Italian fattening pigs and, to a lesser extent, in veal calf holdings.

## Introduction

Carbapenemase-producing Enterobacterales (CPE) are considered a major public health issue and Class D carbapenemases are the main causes of carbapenem resistance among *A. baumannii* and Enterobacterales isolated from human infections in many countries (Pitout et al., 2019). These enzymes show activity against amino-, carboxy-, and ureidopenicillins and narrow-spectrum cephalosporins (e.g., cephalothin), and they also have limited activities against broad-spectrum cephalosporins (especially ceftazidime) and most beta-lactam inhibitors (Pitout et al., 2019). The group II OXA carbapenemases is composed by the OXA-48-related variants (OXA-48-like beta-lactamases). The OXA-181, differing by four aminoacid substitutions from OXA-48, is the most common OXA-48-like variant so far identified (Pitout et al., 2019).

Mobilization and transfer of OXA-48-like beta-lactamases from the chromosome of *Shewanella* spp. onto conjugative plasmids of other bacterial species has been likely associated to mobile genetic elements (MGEs), as the composite transposon Tn1999, responsible for the spread of this antimicrobial resistance (AMR) gene to Enterobacterales (Potron et al., 2011b). The current international spread of *bla*_OXA-181_ among Enterobacterales has been mainly associated to the insertion of the element IS*Ecp1*, which is situated within Tn2013 upstream of *bla*_OXA-181_ and the ΔlysR-Δere (downstream *bla*_OXA-181_), located on different plasmids mainly belonging to the ColE2, IncX3, IncN1, and IncT replicon types (Potron et al., 2011a). The presence of IS*Ecp1* has been previously reported to facilitate the acquisition of ESBL genes such as *bla*_CTX-M-15_ (Poirel et al., 2008).

In humans, *bla*_OXA-181_ was first described in Enterobacterales isolates from Indian patients (Potron et al., 2011a) and over the next 3 years (2012–2014), it spread also in Europe, Asia, and Africa (Ruppé et al., 2014; Lunha et al., 2016; Ouédraogo et al., 2016), with the majority of isolates being co-producers of NDM-1 and having a travel history to the Indian sub-continent (Ruppé et al., 2014). Indeed, the Indian sub-continent has been considered a reservoir of all types of carbapenemases, including OXA-181 (Nordmann and Poirel, 2014). Since 2014, OXA-181 was further detected worldwide (Rojas et al., 2017). OXA-48-like carbapenemases, although their true burden is likely underestimated at global level due to the difficulty in their detection (Boyd et al., 2022), already represent a great clinical and public health concern, since they are the cause of considerable case-fatality rates in infected patients (Bakthavatchalam et al., 2016).

In Europe, the genetic environment (IncT-type plasmid) of a *bla*_OXA-181_ was described in a *Citrobacter freundii* human isolate co-producing NDM-1 (Villa et al., 2013). In Italy, one OXA-181-producing *E.coli* co-harboring *bla*_CTX-M-15_, *bla*_CMY-2_, and *qnrS1* genes was described from the rectal swab of a human patient (Piazza et al., 2018), and one from a pediatric patient, associated with *bla*_NDM-5_ (Marchetti et al., 2020). The *E. coli* IncX3 plasmid named pKP_BO_OXA181 from an Italian patient was also submitted to Genbank in 2017 (GenBank accession number MG228426).

In companion animals, *bla*_OXA-181_ was recently reported in extraintestinal Pathogenic *Escherichia coli* in a dog from Portugal (Brilhante et al., 2020) and in *E. coli* isolated in rectal swabs from hospitalized dogs in Switzerland (Nigg et al., 2019). To date, indeed, there are only very limited findings on OXA-48-and OXA-48-like producing *Enterobacteriaceae* in livestock. In Egypt, *bla*_OXA-48_ and *bla*_OXA-181_ were identified in different *E. coli* isolates from healthy dairy cattle (Braun et al., 2016).

*bla*_OXA-181_ was detected with *bla*_CMY-2_, *qnrS1*, *arm*A, and *mcr*-1 in *E. coli* associated to diarrhea and oedema disease in two pigs sampled in Italy (Pulss et al., 2017). However, in this latter case description, there is no further information on the presence and prevalence of *bla*_OXA-181_ in related commensal *E.coli*, nor tracing back investigations have been performed to hypothesize possible transmission routes of *bla*_OXA-181_.

The aims of this study are: (i) to describe the results of the EU Harmonized AMR Monitoring program conducted in 2021 of OXA-181-(and one OXA-48-) producing *E. coli* isolated mainly from caecal samples in pigs and, to a lesser extent in bovines <12 months, and the tracing back investigations conducted after the first positive case at slaughter; (ii) to investigate by WGS analysis the molecular mechanisms responsible for the mobilization and spread of *bla*_OXA-181_ gene in food-producing animals and in-contact humans, performing *in silico* typing, determination of *bla*_OXA-48_-like genetic context and full reconstruction of the plasmids where the *bla*_OXA-181_ gene(s) were located; and (iii) to study the genomic epidemiology of *bla*_OXA-48_-like *E. coli* and assess the genetic relationships among isolates from animal and human hosts carriers detected at the holding of origin of the positive slaughter batch and at epidemiologically related pig and dairy holdings.

## Materials and methods

### Sampling and isolate identification

In the frame of the Harmonized European Monitoring for AMR (EU Decision 2013/652^1^ and 2020/1729^2^) conducted in Italy in 2021, 301 caecal content samples from fattening pigs and 310 caecal content samples from bovine animals under 1 year of age were collected. These samples obtained from randomly selected epidemiological units at slaughterhouses, were stratified according to EFSA Technical specifications^3^ in Italian regions accounting for >80% on the national throughput of pork and veal meat. Epidemiological unit for fattening pigs and bovines <12 months was the slaughter batch (animals raised together in the same holding of origin).

Beside the results of the survey at slaughter, here we report the results of samples taken by the Competent Authorities (Veterinary Services) in the context of the tracing back and sampling activity in relation to the first positive fattening holding. In this context, fresh fecal samples from the floor of the boxes (of the holdings) where the animals were kept, were taken at (a) the fattening holding of origin of the first positive slaughter batch; (b) the holding providing weaners to the above fattening holding; and (c) one epidemiologically related dairy cattle holding. Additionally, two human fecal samples from one worker and the owner at breeding holding (holding b) only, were voluntary made available through the Local Health Competent Authorities (after giving informed consent to participate in the study).

According to the protocol of the European Union Reference Laboratory for Antimicrobial Resistance,^4^ all samples were cultured by the isolation method specific for CPE using selective media for CR *E. coli.* Briefly, caecal or fecal samples (depending on whether it was monitoring at slaughter or tracing back investigation), were cultured in buffered peptone water at 1:10 (w/v) concentration and incubated at 37°C overnight. 10 μl of the pre-enrichment broth was plated in selective media bi plate for CPE (CHROMID™CARBA SMART; bioMérieux) and incubated overnight at 37°C. One suspected OXA-48-like-producing colony from each sample was isolated on blood agar plates and identified as *bla*_OXA-48_(like)-positive *E. coli* by specific screening PCRs (Poirel et al., 2011). Confirmed *bla*_OXA-48_(like)-positive *E. coli* isolates were in-depth characterized.

### Antimicrobial susceptibility testing

Antimicrobial susceptibility testing was performed as minimum inhibitory concentration (MIC) determination by broth microdilution, using the EU consensus 96-well microtiter plates (Trek Diagnostic Systems, Westlake, OH, United States). The following antimicrobials were tested, as reported in the EU Decision 2020/1729/EU: amikacin, ampicillin, azithromycin, cefotaxime, ceftazidime, chloramphenicol, ciprofloxacin, colistin, gentamicin, meropenem, nalidixic acid, sulfamethoxazole, tetracycline, tigecycline, trimethoprim (first panel) and cefepime, cefotaxime, cefotaxime + clavulanic acid, cefoxitin, ceftazidime, ceftazidime + clavulanic acid, ertapenem, imipenem, meropenem, and temocillin (second panel). Dilution ranges and interpretation of MIC values using epidemiological cutoffs (ECOFFs) were performed according to the EU Decision 2020/1729/EU and to the EFSA manual published in 2021 [European Food Safety Authority (EFSA) et al., 2021]. For carbapenems and temocillin, results were also interpreted according to EUCAST^5^ clinical breakpoints (Table 1). *Escherichia coli* ATCC 25922 was used as quality control strain.

**Table 1:** Epidemiological cutoffs (ECOFFs) and clinical breakpoints for carbapenems and temocillin, with number (nR) and percentage (%) of *E. coli* resistant isolates from the survey at slaughter.

	**R (ECOFF)**	**R (CB)**	nR/tot (%) ECOFF	nR/tot (%) CB
**ETP (ertapenem)**	>0,06	>0,5	25/25**(100%)**	7/25 (28%)
**IMI (imipenem)**	>0,5	>4	5/25 (20%)	5/25(20%)
**MER (meropenem)**	>0,125	>8	13/25 (52%)	0/25 (0%)
**TRM (temocillin)**	>16	>16	25/25**(100%)**	25/25**(100%)**

R, resistance; CB, Clinical breakpoint; ECOFF, Epidemiological cut-off; nR/tot, number of resistant isolates/total of isolates from the survey at slaughter.

The results of AST were further compared with the AMR genotypes to determine if the phenotypic resistance patterns were confirmed by the presence of the corresponding AMR genes.

### Library preparation and whole genome sequencing

The *bla*_OXA-48_(like)-positive isolates obtained from pigs at slaughter and from the tracing back activity after the first positive fattening holding, were investigated by WGS analysis.

DNA extraction and library preparation were performed according to Alba et al. (2021). Briefly, genomic DNA was extracted using the QIAamp DNA Mini Kit (Qiagen, Hilden, Germany) following the manufacturer’s protocol. Libraries for short reads pair-end sequencing were prepared using the NexteraXT DNA library preparation kit (Illumina, Inc., San Diego, CA, United States) and sequenced on an Illumina platform (MiSeq).

In parallel, libraries of 16 selected isolates, with five of them obtained from pigs at slaughter (Table 2) and 11 isolated in the frame of the tracing back investigations (Table 3) were prepared with the rapid barcoding kit (SQK-RBK004) and sequenced using the nanopore-based MinION device (ONT; Branton et al., 2008).

**Table 2:** Metadata and genomic characteristics of the OXA-48(like) producing *E. coli* obtained from the samples collected from the survey at slaughter.

**Isolate ID**	**Region**	**Host**	**Production type**	**ST**	**Serotype**	**Carbapenemase type**	**AMR genes**	**Inc Plasmid replicons**	**Chromosomal Point Mutations**	**Phenotypic AMR pattern**	**Sample accession number**
21019054–1^*^	C	Pig	Fattening	48	O70:H11	OXA-181	*aad*A2^§^ *bla*_OXA-181_ *bla*_TEM-1B_ *cml*A1^§^ *dfr*A12 *mef*(B) *sul*3 *tet*(A)^§^ *tet*(M)^§^	IncX3 IncY		CHL, TMP, SMX, TET, AMP, ETP, FEP, and TRM	ERS12773674
21034263–1*	D	Pig	Fattening	5229	-:H51	OXA-181	*aac*(3)-IId^§^ *aad*A17^§^ *ant*(3″)-Ia^§^ *bla*_OXA-181_ *cml*A1^§^ *dfr*A12 *flo*R^§^ *lnu*(F) *sul*2 *sul*3 *tet*(A)^§^ *tet*(M) ^§^	IncFIC(FII) IncX1	*gyr*A p.D87N *par*C p.S80I	CHL, GEN, NAL, TMP, SMX, TET, AMP, CIP, ETP, MER, and TRM	ERS12773675
2105491–1*	C	Pig	Fattening	10	O101; O9a:H9	OXA-181	*aph*(3″)-Ib *aph*(6)-Id *bla*_OXA-181_ *bla*_TEM-1B_ *dfr*A5 *qnr*S1 *sit*ABCD^§^ *sul*2	IncFII IncX3		TMP, SMX, AMP, CIP, MER, ETP, IMI, FOT, FEP, and TRM	ERS12773676
21055959–1*	D	Pig	Fattening	5229	-:H51	OXA-181	*aac*(3)-IId^§^ *aad*A17^§^ *ant*(3″)-Ia^§^ *bla*_OXA-181_ *bla*_TEM-1B_ *cml*A1^§^ *dfr*A12 *flo*R^§^ *lnu*(F) *qnr*S1 *sul*2 *sul*3 *tet*(A)^§^ *tet*(M)^§^	IncFIC(FII) IncX3	*gyr*A p.D87N *par*C p.S80I	CHL, GEN, NAL, TMP, SMX, TET, AMP, CIP, ETP, IMI, MER, FEP, FOT, FOX, and TRM	ERS12773677
21066975–1*	C	Pig	Fattening	5229	-:H51	OXA-181	*aac*(3)-IId^§^ *aad*A17^§^ *ant*(3″)-Ia^§^ *bla*_OXA-181_ *bla*_TEM-1B_ *cml*A1^§^ *dfr*A12 *flo*R^§^ *lnu*(F) *sul*2 *sul*3 *tet*(A)^§^ *tet*(M)^§^	IncFIC(FII) IncX1	*gyr*A p.D87N *par*C p.S80I	CHL, GEN, NAL, TMP, SMX, TET, AMP, CIP, ETP, FOT, and TRM	ERS12773678
21076969–1	C	Pig	Fattening	3489	O153:H25	OXA-181	*aac*(3)-IId^§^ *aph*(3″)-Ia *bla*_CTX-M-55_ *bla*_OXA-181_^#^ *cml*A1^§^ *dfr*A12 *flo*R^§^ *fos*A3 *lnu*(F)^§^ *qnr*S1 *rmt*B *sul*2 *sul*3 *tet*(A)^§^ *tet*(M)^§^	IncFIB(AP001918) IncFIC(FII) IncFII(pHN7A8) IncX3^#^	*par*C p.S80I	CHL, GEN, NAL, TMP, SMX, TET, AMP, CIP, AMK, ETP, MER, FEP, FOT, TAZ, and TRM	ERS12773679
21080958–1	C	Pig	Fattening	5229	-:H51	OXA-181	*aac*(3)-IId^§^ *bla*_OXA-181_ *cml*A1^§^ *dfr*A12 *flo*R^§^ *lnu*(F) *sul*2 *sul*3 *tet*(A)^§^ *tet*(M) ^§^	IncFIB(AP001918) IncFIC(FII) IncX1	*gyr*A p.D87N *par*C p.S80I	CHL, GEN, NAL, TMP, SMX, TET, AMP, CIP, ETP, IMI, MER, FOT, and TRM	ERS12773680
21088819–1	D	Pig	Fattening	117	-:H4	OXA-181	*aac*(3)-IId^§^ *aph*(3″)-Ib *aph*(6)-Id *bla*_OXA-181_^#^ *bla*_TEM-1B_ *dfr*A17 *qnr*S1 *sit*ABCD^§^ *tet*(A) ^§^	IncFIB(AP001918) IncFIC(FII) IncX3^#^	*gyr*A p.S83L	GEN, NAL, TMP, TET, AMP, CIP, ETP, and TRM	ERS12773681
21094270–1	C	Bovine	Beef	5229	-:H51	OXA-181	*aac*(3)-IId^§^ *aph*(3″)-Ia *bla*_OXA-181_ *cml*A1^§^ *dfr*A12 *flo*R^§^ *lnu*(F) *qnr*S1 *sul*2 *sul*3 *tet*(B) *tet*(M)^§^	IncFIB(AP001918) IncFIC(FII) IncX3	*gyr*A p.D87N *par*C p.S80I	TMP, SMX, TET, AMP, CIP, FOT, ETP, MER, TAZ, and TRM	ERS12773682
21097167–1	C	Pig	Fattening	542	-:H38	OXA-181	*aad*A2^§^ *aph*(6)-Id *aph*(3″)-Ib^§^ *bla*_OXA-181_^#^ *bla*_TEM-1B_ *cml*A1^§^ *dfr*A1^§^ *sul*1^§^ *sul*3 *tet*(B)	IncFIB(pHCM2) IncFII(pCRY)^#^ IncX1 IncY		CHL, TMP, SMX, TET, AMP, MER, ETP, FEP, and TRM	ERS12773683
21100090–1	C	Pig	Fattening	410	-:H9	OXA-181	*aac*(3)-IVa^§^ *aad*A2^§^ *aph*(3″)-Ia *aph*(3″)-Ib^§^ *aph*(4)-Ia *aph*(6)-Id *bla*_CARB-2_ *bla*_OXA-181_ *cml*A1^§^ *dfr*A16 *flo*R^§^ *sul*3 *tet*(A)^§^	IncFIA(HI1) IncI1-I(Alpha) IncR IncX1 IncY	*gyr*A p.D87N *par*C p.S80I *par*E p.S458A	CHL, GEN, NAL, TMP, SMX, TET, AMP, CIP, ETP, MER, FEP, and TRM	ERS12773684
21100098–1	D	Pig	Fattening	410	O159:H28	OXA-181	*aac*(3)-IIa *aad*A2^§^ *aph*(3″)-Ib *aph*(3″)-Ia *bla*_OXA-181_ *bla*_TEM-1A_ *cat*A1^§^ *cml*A1^§^ *dfr*A12 *flo*R^§^ *mef*(C)^§^ *mph*(A)^§^ *mph*(G) *qnr*S1 *sul*2^§^ *tet*(B) *tet*(M) ^§^	IncFIB(AP001918) IncFIC(FII) IncI1-I(Alpha) IncX3	*gyr*A p.D87N *par*C p.S80I *par*E p.S458A	CHL, GEN, AZI, NAL, TMP, SMX, TET, AMP, CIP, ETP, and TRM	ERS12773685
21102457–1	C	Pig	Fattening	38	O86:H30	OXA-48	*aac*(3)-IId^§^ *aad*A5 *bla*_OXA-48_ *bla*_TEM-1B_ *cat*A1^§^ *dfr*A17 *mph*(A)^§^ *sul*1^§^ *tet*(D)		*gyr*A p.S83A	GEN, AZI, NAL, TMP, SMX, TET, AMP, CIP, MER, ETP, IMI, FEP, FOT, and TRM	ERS12773686
21109909–1	C	Pig	Fattening	5229	O76:H51	OXA-181	*aac*(3)-IId^§^ *aph*(3″)-Ia *bla*_OXA-181_^#^ *bla*_TEM-1B_ *cat*A2^§^ *cml*A1^§^ *dfr*A12 *flo*R^§^ *lnu*(F) *sul*2 *sul*3 *tet*(A)^§^ *tet*(M)^§^	IncFIB(AP001918) IncFIC(FII) IncX3^#^	*gyr*A p.D87N *par*C p.S80I	CHL, GEN, NAL, TMP, SMX, TET, AMP, CIP, ETP, FOT, and TRM	ERS12773687
21110411–1	A	Pig	Fattening	/	-:H10	OXA-181	*aac*(3)-IIa *aph*(3″)-Ib^§^ *aph*(6)-Id *bla*_DHA-1_ *bla*_OXA-181_^§^ *bla*_TEM-1B_ *cat*A1^§^ *dfr*A17 *dfr*A1^§^ *flo*R^§^ *mph*(A)^§^ *qnr*B4 *sul*1^§^ *sul*3	IncFII IncX3 IncY		CHL, GEN, AZI, TMP, SMX, AMP, CIP, ETP, FOT, FOX, TAZ, and TRM	ERS12773688
21112463–1	C	Pig	Fattening	5229	-:H45	OXA-181	*aac*(3)-IId^§^ *bla*_CMY-2_ *bla*_OXA-181_ *bla*_TEM-30_ *cml*A1^§^ *dfr*A12 *flo*R^§^ *lnu*(F) *sul*2 *sul*3 *tet*(A)^§^ *tet*(M)^§^	IncFIB(AP001918) IncFIC(FII) IncFII(pCoo) IncI1-I(Alpha) IncX1	*gyr*A p.D87N *par*C p.S80I	CHL, GEN, NAL, TMP, SMX, TET, AMP, CIP, MER, ETP, IMI, FEP, FOT, FOX, TAZ, and TRM	ERS12773689
21112465–1	E	Pig	Fattening	542	O167:H20	OXA-181	*aac*(3)-IVa^§^ *aad*A2^§^ *aph*(3″)-Ia *aph*(4)-Ia *bla*_OXA-181_^#^ *bla*_TEM-1B_ *cml*A1 *dfr*A1^§^ *flo*R^§^ *mph*(B) *sul*1^§^ *sul*3 *tet*(A)^§^ *tet*(M)^§^	IncFIB(AP001918) IncFIC(FII) IncX1 IncX3^#^		CHL, GEN, NAL, TMP, SMX, TET, AMP, CIP, MER, ETP, FEP, FOX, and TRM	ERS12773690
21116039–1	B	Bovine	Beef	5229	-:H9	OXA-181	*aac*(3)-IId^§^ *aph*(3″)-Ia *bla*_OXA-181_ *bla*_TEM-1B_ *cml*A1^§^ *dfr*A12 *flo*R^§^ *lnu*(F) *sul*2 *sul*3 *tet*(A)^§^ *tet*(M) ^§^	IncFIB(AP001918) IncFIC(FII) IncX1	gyrA p.D87N parC p.S80I	CHL, GEN, NAL, TMP, SMX, TET, AMP, CIP, MER, ETP, FEP, and TRM	ERS12773691
21122716–1	C	Pig	Fattening	5229	-:H51	OXA-181	*aac*(3)-IId^§^ *bla*_OXA-181_ *bla*_TEM-1B_ *cml*A1^§^ *dfr*A12 *flo*R^§^ *lnu*(F) *sul*2 *sul*3^§^ *tet*(A)^§^ *tet*(M) ^§^	IncFIB(AP001918) IncFIC(FII) IncX1	*gyr*A p.D87N *gyr*A p.S83L *par*C p.S80I	CHL, GEN, NAL, TMP, SMX, TET, AMP, CIP, MER, ETP, and TRM	ERS12773692
21126335–1	C	Bovine	Beef	542	O163:H20	OXA-181	*aph(3″)-Ib**^§^* *aph(6)-Id bla_OXA-181_ bla_TEM-1B_ catA1**^§^* *dfrA1**^§^* *floR**^§^* *lnu(G) qnrS1 sul3 tet(B)*	IncFIB(AP001918) IncFIC(FII) IncX3 IncY		CHL, NAL, TMP, SMX, TET, AMP, CIP, ETP, FEP, and TRM	ERS12773693
21127652–1	C	Pig	Fattening	218	-:H38	OXA-181	*aad*A2^§^ *aph*(3″)-Ib^§^ *aph*(6)-Id *bla*_OXA-181_^#^ *bla*_TEM-1B_ *cml*A1^§^ *dfr*A12 *sul*3 *tet*(B)	IncR IncX3^#^		CHL, TMP, SMX, TET, AMP, ETP, and TRM	ERS12773694
21130011–1	D	Bovine	Beef	10	O13/O129;O13/O135:H48	OXA-181	*aac*(3)-IIa *aac*(3)-IVa^§^ *ant*(3″)-Ia^§^ *aph*(3″)-Ib^§^ *aph*(4)-Ia *aph*(6)-Id *bla*_CTX-M-1_ *bla*_OXA-181_ *flo*R^§^ *lnu*(G) *mph*(A)^§^ *qnr*S1 *sul*1^§^ *tet*(A)^§^ *tet*(B) *tet*(M)^§^	IncFIA(HI1) IncFIB(AP001918) IncFIC(FII) IncHI1A IncHI1B(R27) IncX3		CHL, GEN, SMX, TET, AMP, CIP, ETP, FEP, FOT, TAZ, and TRM	ERS12773695
21133286–1	B	Pig	Fattening	48	O8:H11	OXA-181	*aac*(3)-IVa^§^ *aph*(3″)-Ia *aph*(4)-Ia *bla*_OXA-181_ *bla*_TEM-1B_ *flo*R^§^ *qnr*S1 *tet*(A)^§^ *tet*(M)^§^	IncFIB(AP001918) IncFIC(FII) IncX1 IncX3		CHL, GEN, NAL, TET, AMP, CIP, ETP, and TRM	ERS12773696
21135758–1	B	Pig	Fattening	7461	O112ab:H38	OXA-181	*bla*_OXA-181_ *qnr*S1	IncX3	gyrA p.S83L	NAL, AMP, CIP, MER, ETP, and TRM	ERS12773697
21137340–1	B	Pig	Fattening	101	O82:H8	OXA-181	*aph*(3″)-Ib^§^ *aph*(6)-Id^§^ *bla*_OXA-181_ *dfr*A1 *qnr*S1 *sit*ABCD^§^ *sul*2 *tet*(B)	IncB/O/K/Z_2 IncFIA IncFIB(AP001918) IncFIC(FII) IncFII(pCTU2) IncX3		TMP, SMX, TET, AMP, CIP, ETP, and TRM	ERS12773698

* ONT-sequenced.

# located on the same contig.

§ ResFinder identity or coverage < 100%.

CHL, chloramphenicol; GEN, gentamicin; AZI, azithromycin; NAL, nalidixic acid; TMP, trimethoprim; COL, colistin; SUL, sulfamethoxazole; TET, tetracycline; AMP, ampicillin; CIP, ciprofloxacin; AMK, amikacin; MER, meropenem; FOT, cefotaxime; TAZ, ceftazidime; TGC, tigecycline; ETP, ertapenem; IMI, imipenem; FEP, cefepime; FOX, cefoxitin; and TRM, temocillin.

**Table 3:** Metadata and genomic characteristics of the OXA-181 producing *Escherichia coli* obtained from the samples collected in the context of the tracing- back activities after the first OXA-181-producing *E. coli* isolate detection.

**Isolate ID**	**Host**	**Production type**	**Sampling site**	**ST**	**Serotype**	**AMR genes**	**Inc plasmid replicons**	**Sample accession number**
21019054–1	Pig	Fattening	Slaugtherhouse	48	O70:H11	*aad*A2^§^ *bla*_OXA-181_ *bla*_TEM-1B_ *cml*A1^§^ *dfr*A12 *mef*(B) *sul*3 *tet*(A)^§^ *tet*(M)^§^	IncX3 IncY	ERS12773674
21041921–5	Pig	Fattening	Pen 1 (box 5)	5229	O76:H51	*aac*(3)-IId^§^ *ant*(3″)-Ia^§^ *aph*(3″)-Ia^§^ *bla*_OXA-181_ *bla*_TEM-1B_ *flo*R^§^ *lnu*(F) *sul*2 *sul*3 *tet*(A)^§^	IncFIC(FII) IncX1	ERS12841964
21041921–11	Pig	Fattening	Pen 1 (box 11)	5229	-:H51	*aad*A2^§^ *bla*_CTX-M-1_ *bla*_OXA-181_ *bla*_TEM-1B_ *cml*A1^§^ *dfr*A12 *flo*R^§^ *mph*(A)^§^ *sul*2 *sul*3 *tet*(M)^§^	IncFIC(FII) IncX1	ERS12841965
21058774–1	Pig	Breeding	Gestation Unit	224	O8:H23	*aac*(3)-IIa *aac*(3)-IVa^§^ *aad*A5 *aph*(3″)-Ia *aph*(4)-Ia *arm*A *bla*_OXA-181_ *bla*_TEM-1A_ *dfr*A17 *mef*(C)^§^ *mph*(G) *sul*1^§^	IncFII IncI1-I(Alpha) IncX1 IncY	ERS12841966
21058774–11	Pig	Breeding	Farrowing Unit (pen)	5229	O76:H51	*aac*(3)-IId^§^ *ant*(3″)-Ia^§^ *aph*(3″)-Ia *bla*_OXA-181_ *bla*_TEM-1B_ *flo*R^§^ *lnu*(F) *sul*2 *sul*3 *tet*(A)^§^	IncFIC(FII) IncX1	ERS12841967
21058774–16	Pig	Breeding	Weaner Unit “A”	1494	O9a:-	*aac*(3)-IId^§^ *aad*A2§ *bla*_OXA-181_ *bla*_TEM-1B_ *dfr*A12 *flo*R^§^ *lnu*(F)^§^ *sul*3 *tet*(A)^§^ *tet*(B) *tet*(M)^§^	IncFIA(HI1) IncFIB(K) IncFII(pCRY) IncR IncX1	ERS12841968
21058774–18	Pig	Breeding	Weaner Unit “B”	5229	O76:H51	*aac*(3)-IId^§^ *ant*(3″)-Ia^§^ *aph*(3″)-Ia *bla*_OXA-181_ *bla*_TEM-1B_ *flo*R^§^ *lnu*(F) *sul*2 *sul*3 *tet*(A)^§^	IncFIB(AP001918) IncFIC(FII) IncX1	ERS12841969
21058774–20	Pig	Breeding	Weaner Unit “C”	5229	O76:H51	*aac*(3)-IId^§^ *ant*(3″)-Ia^§^ *aph*(3″)-Ia *bla*_OXA-181_ *bla*_TEM-1B_ *flo*R^§^ *lnu*(F) *sul*2 *sul*3 *tet*(A)^§^	IncFIC(FII) IncX1	ERS12841970
21058774–21	Pig	Breeding	Finisher Unit	5229	O76:H51	*aac*(3)-IId^§^ *ant*(3″)-Ia^§^ *aph*(3″)-Ia *bla*_OXA-181_ *bla*_TEM-1B_ *flo*R^§^ *lnu*(F) *sul*2 *sul*3 *tet*(A)^§^	IncFIC(FII) IncX1	ERS12841971
21067608–2	Human	N.A.	N.A.	5229	O101:H9	*aac*(3)-IId^§^ *aad*A5 *aph*(3″)-Ib *aph*(6)-Id *bla*_OXA-181_ *bla*_TEM-1B_ *cat*A1^§^ *dfr*A17 *flo*R^§^ *mph*(A)^§^ *sul*1^§^ *sul*2 *tet*(B)	IncFII IncX1	ERS12841972
21067608–3	Human	N.A.	N.A.	744	O76:H51	*aac*(3)-IId^§^ *ant*(3″)-Ia^§^ *aph*(3″)-Ia *bla*_OXA-181_ *bla*_TEM-1B_ *flo*R^§^ *lnu*(F) *sul*2 *sul*3	IncFIC(FII) IncX1	ERS12841973
21067610–3	Bovine	Dairy		5229	O76:H51	*aac*(3)-IId^§^ *ant*(3″)-Ia^§^ *aph*(3″)-Ia *bla*_OXA-181_ *bla*_TEM-1B_ *flo*R^§^ *lnu*(F) *sul*2 *sul*3 *tet*(A)^§^	IncFIC(FII) IncX1	ERS12841974

§ ResFinder identity or coverage < 100%.

### Bioinformatics analysis

Illumina raw reads were analyzed using an internal pipeline for assembly, which includes the following tools: FastQC,^6^ Trimmomatic (Bolger et al., 2014), Spades (Bankevich et al., 2012), and Quast (Gurevich et al., 2013). Molecular characterization was performed on all the assembled genomes with the MLST tool^7^ for Multilocus Sequence Typing (MLST) analysis and with the ABRicate tool^8^ using the Genomic Epidemiology (CGE) databases (last update 22/04/2022) of ResFinder (with a threshold of 95% for coverage and identity) and PointFinder^9^ for the detection of the genetic basis of AMR to confirm the AMR phenotypes, PlasmidFinder^10^ for plasmid replicon typing, SerotypeFinder^11^ for *in silico* serotyping and VirulenceFinder^12^ for virulotyping. Additionally, the VF database^13^ was used for virulence gene detection.

For the samples subjected to both short and long reads sequencing, high accuracy basecalling was performed on long-reads obtained from the MinION device according to the ONT workflow. A hybrid (Illumina-ONT) assembly was performed using the Unicycler pipeline (Wick et al., 2017) with the default parameters (Diaconu et al., 2020). Assemblies from the OXA-181 producing isolates recovered during the monitoring were compared using the Mashtree algorithm (Katz et al., 2019). The assemblies obtained were annotated using the Bakta tool (Schwengers et al., 2021). Additionally, a manual curation for the obtained annotation was also performed. All the obtained plasmid sequences were compared calculating the average nucleotide identity (ANI) for the alignment coverage and identity, using the BLAST algorithm (Goris et al., 2007; Supplementary Table 1).

Representative hybrid assemblies of the different plasmids harboring *bla*_OXA-181_, were compared by BLAST, using both the stand alone tool and the on-line tool against the nr/nt database. After that, assemblies of the IncX3 plasmids detected in this study were compared with selected IncX3 plasmid sequences obtained from publicly available databases with more than 80% of identity: FDAARGOS_433 (CP023897) from Canada, pAMA1167 (CP024806) from Denmark, pOXA 181_29144 (KX523903) from Czech Republic, pSTIB_IncX3_OXA_181 = IncX3_OXA_181 (MG570092) from Lebanon, pOXA181 IHIT35346 (KX894452) from Italy, pKBN10P04869C (CP026476) from South Korea, pKP_BO_OXA181 (MG228426) from Italy, and NZ_CP040399.1 from India. The complete sequence of the IncX3 plasmid pMOL4791 from sample ID 21054791 (accession number ERS12413439) was used as reference.

Similarly, our IncX1 plasmid sequences were compared with publicly available databases using the IncX1 plasmid pMOL6975 from sample ID 21066975 (accession number ERS12413440). Results were represented using BRIG (Alikhan et al., 2011) or using the R^14^ package ggplot2 version 3.3.6.9000.

## Results

### OXA-48-like producing *Escherichia coli* prevalence at slaughterhouse

Overall, *n* = 21 out of 301 caecal samples of fattening pigs (6.98%; 95% CI 4.37–10.47%) and *n* = 4 out of 310 bovines <12 months (1.29%; 95% CI 0.35- 3.27%) resulted positive to OXA-48-like-producing *E. coli* (*n* = 24 OXA-181, *n* = 1 OXA-48).

### Antimicrobial susceptibility testing

The complete results and interpretation of AST performed on the 25 OXA-48-like-producing *E. coli* obtained from the survey at slaughter, are reported in Supplementary Table 1 and Table 2. The isolates presented a MDR resistance profile ranging from a pattern of minimum four resistances (beta-lactams, carbapenems, fluoroquinolones, and trimethoprim, 1/25, 4.0%) up to a pattern of eight resistances (carbapenems, fluoroquinolones, aminoglycosides, sulphonamides, tetracyclines, trimethoprim, phenicols, all beta-lactams or macrolides 3/25, 12.0%). As for the carbapenem resistance phenotypes, all of them showed microbiological resistance to ertapenem and 13/25 (52%) to meropenem, while 5/25 (20%) showed microbiological and clinical resistance to imipenem. All of them were also microbiologically and clinically resistant to the semi-synthetic betalactam antibiotic temocillin (Table 1). Additionally, 17/25 (68%) isolates, were concomitantly resistant to aminoglycosides, with all of them showing resistance to gentamicin and one also to amikacin (ID 21076969–1). Moreover, some isolates were also resistant to certain Highest Priority Critically Important antimicrobials (HPCIAs), with almost (22/25, 88%) all being resistant to ciprofloxacin and 3/25 (12%) also to azithromycin. Four isolates were Extended-Spectrum Cephalosporins (ESC)-R, being resistant to cefotaxime and ceftazidime, while elevated MICs for cefotaxime slightly above the CUTOFF (mode = 0.5 mg/L) were observed in other five isolates.

### Bionformatics analysis of OXA-48-(like)-producing *Escherichia coli* from the survey at slaughter (short-read sequencing)

Genomic characterization of the 24 OXA-181 and one OXA-48 producing isolates indicated that the OXA-181-producing isolates presented a high degree of Sequence Types (STs) diversity, although nine out of 25 isolates belonged to ST5229.

According to the results of SerotypeFinder, the isolates belonged to different serotypes (Table 2), and none of them belonged to the most relevant serogroups associated to Shiga-Toxin *E. coli* (STEC) infections in humans. Two isolates (IDs 21058774–1, 21133286–1) belonged to the serogroup O8, which is frequently found in the intestinal pathogenic *E. coli* (inPEC) pathotype implicated in post-weaning diarrhea in swine. However, in these isolates, the genes encoding the fimbriae F4, F5, F6, and F41 and F4, F18ac, which are typical of this pathotype (Luppi, 2017; Yang et al., 2019), were lacking.

As for the results of the virulence-associated determinants detected by VirulenceFinder and VF database, almost all isolates harbored the curli fimbriae (*csgA*) and the type 1 fimbriae (*fim*H), while only some of them presented at least one of the following virulence genes: *fim*A, *ast*A encoding the enteroaggregative *E. coli* heat-stable enterotoxin 1 (EAST1), *pap*C, *iuc*D, *pic*, and *hly*F. Of these, only one isolate (ID 21088819) harbored all the above mentioned genes except for *fim*A. However, none of these genes indicated the presence of a specific *E. coli* pathotype. Three isolates, 21054791, 21088819, and 21137340, presented the gene *sit*ABCD, and one of them (21088819) presented also *kat*P, both responsible for resistance to hydrogen peroxide.

In general, the AMR genotypes were in agreement with the MDR phenotypic resistance patterns (Table 2).

As for the MDR phenotypes including resistance to HPCIAs, two of the four ESC-R isolates, (IDs 21076969 and 21130011) were confirmed ESBL producers, harboring *bla*_CTX-M-55_ and *bla*_CTX-M-1_, respectively, while isolates with IDs 21110411 and 21112463, had an AmpC profile, presenting *bla*_CMY-2_ and *bla*_DHA-1_ genes, respectively. As for the 22 ciprofloxacin resistant isolates, 15 of them presented some chromosomal mutations associated with (fluoro)quinolone resistance, *gyr*A p.D87N or p.S83A, *par*C p.S80I, or *par*E p.S458A. Moreover, 12 isolates presented the transferable quinolone resistance (*qnr*) genes *qnr*S1 and one isolate *qnr*B4. In six cases, these *qnr* genes were found together with chromosomal mutations associated to (fluoro)quinolone resistance. One isolate presented a (fluoro)quinolone resistance phenotype (ciprofloxacin MIC value of 16 mg/L), but did not harbor any related known accessory resistance gene or chromosomal point mutation. The OXA-48-producing isolate presented the following AMR genes profile: *aac*(3)-IId, *aadA5*, *bla*_OXA-48_, *bla*_TEM-1B_, *catA1*, *dfrA17*, *mph*(A), *sul1*, and *tet*(D).

**Figure 1:**
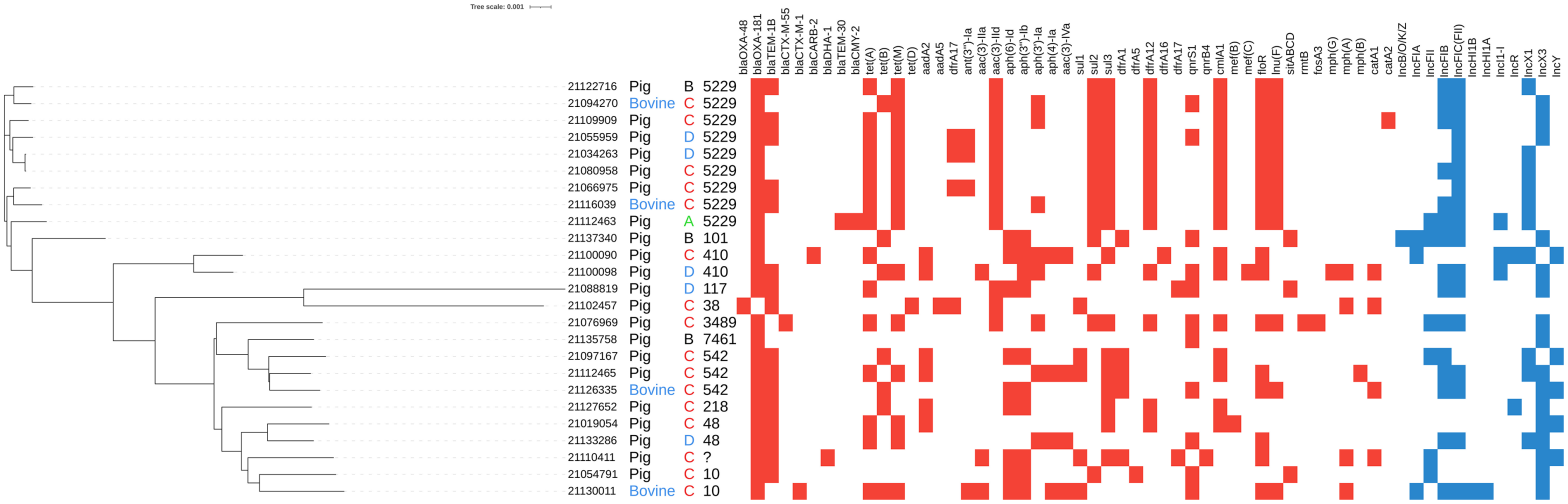
Mash clusterization of the complete genome, resistome and plasmidome of the 25 OXA-181-producing *Escherichia coli* isolated from caecal samples collected at slaughterhouse during the monitoring activities. From left to right: isolate ID, host (pig in black letters, bovine in blue letters), region anonymized and ST. Colored squares indicate the presence of resistance genes (red squares) and plasmid replicons (blue squares).

Additionally, all 17 gentamicin-resistant isolates harbored at least one of the corresponding gentamicin resistance genes as *aac*(3)-IId, *aac*(3)-IVa, and *aac*(3)-IIa. One of them (ID 21076969–1) showing phenotypic resistance to amikacin presented also the corresponding gene *rmt*B, responsible for phenotypic resistance to amikacin and gentamicin (Figure 1; Table 2).

The results of PlasmidFinder analysis revealed the presence of different plasmid replicons (Figure 1; Table 2) with IncX (IncX1 and IncX3) and IncF types being the most represented in the 25 isolates. In five isolates *bla*_OXA-181_ was located on the same contig of the IncX3 replicon and in one isolate, on the same contig of IncFII (Figure 1; Table 2). Differently, the OXA-48-producing isolate (21102457–1) did not contain any plasmid replicon.

The results of the Mash analysis are represented in Figure 1. The Mash clusterization indicated the presence of a non-clonal population of OXA-48-like producing *E. coli* in the dataset analyzed. The clusters were distributed according to the different Clonal Complexes (CCs) and STs. No clear region or host species correlation was observed.

### Bionformatics analysis of OXA-181-producing *Escherichia coli* from tracing-back activities (short-read sequencing)

A total of 11 OXA-181 producing *E. coli* were obtained from 11 samples collected from different sampling sites within the studied holdings in the context of the tracing back activities related to the *E. coli* isolate 21019054–1 detected from the survey at slaughter (Table 3).

In detail, two isolates were obtained from fecal samples collected from the fattening holding of origin, six from different units of the breeding holding providing weaners, one from an epidemiologically related dairy cattle holding, and two from human samples taken from the worker and owner at the breeding holding. As for the results of molecular characterization, eight out of 11 isolates were classified as ST5229 whereas the other ones were classified as ST224, ST1494, and ST744. None of them belonged to ST48, the ST to which the first positive isolate at slaughterhouse belonged. The results of the *in silico* serotype determination were almost in agreement with the STs, being the serotype most represented O76:H51, associated with ST5229.

All presented a MDR gene profile and, at least, two different plasmid types (Table 3).

### Full reconstruction of the *bla*_OXA-181_-carrying plasmids by hybrid (short and long-read) assembly approach

**Figure 2:**
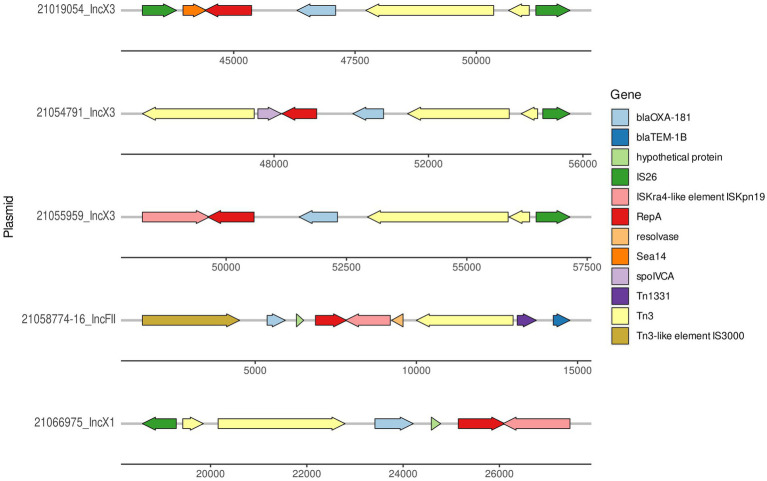
Graphical representation of the region where *bla*_OXA-181_ was located in the three IncX3 plasmids, one representative IncX1 (pMOL6975) from ID21066975 (accession number ERS12413440) and IncFII (21058774–16) plasmids.

The complete sequence of 16 plasmids harboring *bla*_OXA-181_ was obtained through the hybrid (Illumina–ONT) assembly approach. In the studied OXA-181 producing *E. coli* population, *bla*_OXA-181_ was confirmed to be located in different type of plasmids, according to the incompatibility group classification: IncX3 (in three isolates from the survey at slaughter) IncX1 (in two isolates from the survey at slaughter and 10 isolates from tracing-back activities) and IncFII (in one isolate from tracing-back activities). In all the three plasmid types, *bla*_OXA-181_ was part of a transposon with a similar general structure, which in all IncX1 plasmids resulted identical (Figure 2). Coverage and identity values obtained comparing all the 16 fully resolved plasmids are reported in Supplementary Table 1.

**Figure 3:**
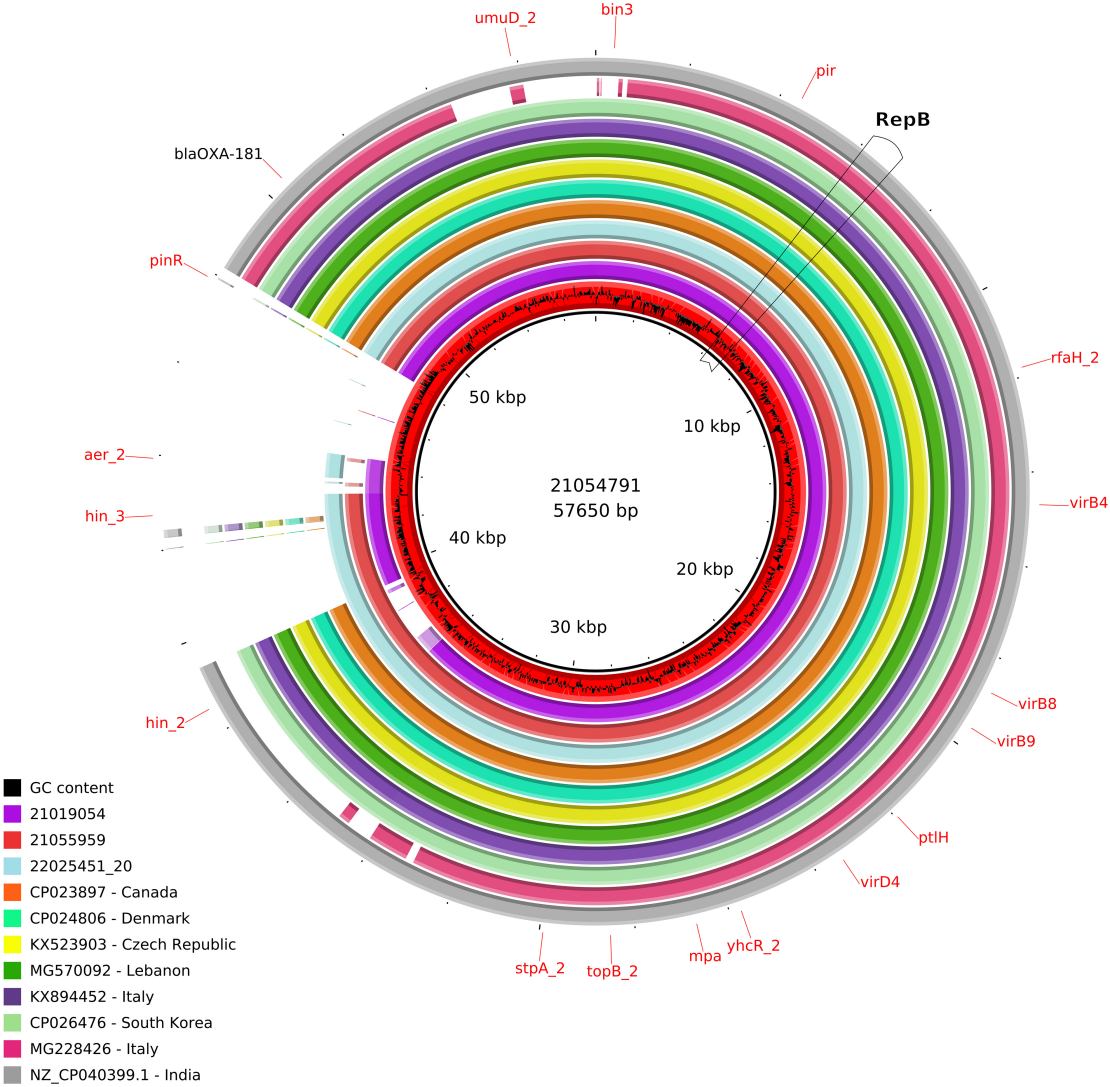
Comparison of the IncX3 plasmids obtained in this study and publicly available IncX3 plasmids containing *bla*_OXA-181_. From the inner circle, the plasmids represented were the following: red: ID21054791 used as reference (accession number ERS12413439), including the CG content graph (sequenced in this study, *Escherichia coli*, caecal content, pig 2021), violet: ID21019054 (sequenced in this study, *E. coli*, pig caecal content 2021), light red: ID21055959 (sequenced in this study, *E. coli*, pig caecal content, 2021), turquoise: ID22025451_20 (sequenced at the Italian NRL-AR; *Citrobacter freundii*, pig feces, 2022), orange: FDAARGOS_433 (CP023897, Canada, *E. coli*, human, 2017), hospital green: pAMA1167_OXA_181 (CP024806, Denmark, *E. coli*, human, 2017), yellow: pOXA_181_29144 (KX523903, Czech Republic, *Klebsiella pneumoniae*, human, 2016), green: pSTIB_IncX3_OXA_181 (MG570092, Lebanon, *E. coli*, human, 2016), purple: pOXA181 IHIT35346 (KX894452, isolated in Italy, *E. coli*, pig, 2016), light green: pKBN10P04869C (CP026476, South Korea, *E. coli*, human, 2017), pink: pKP_BO_OXA181 (MG228426, Italy, *E. coli*, human, 2017), and gray: (NZ_CP040399.1, India, *E. coli*, human, 2016).

The size of the IncX3 plasmids from the ID21019054, ID21055959, and ID21054791 isolates were 51,982; 57,195, and 57,650 bp, respectively. Using the plasmid from ID 21054791 (pMOL4791) as reference, all three resolved plasmids IncX3 harboring *bla*_OXA-181_ were very similar with 100% identity but 90–91% coverage (Supplementary Table 1). The two larger plasmids contained also the resistance gene *qnr*S. The *rep*B gene was located in the region 5931..6944 and *rep*A in 44413..45373, being truncated in the plasmid from ID21054791. Most of the genes found were involved in the stabilization, replication and conjugation of the plasmids, except the resistance genes and the different ISS sequences. The complete sequences of our three IncX3 plasmids from *E. coli* isolates (IDs 21019054, 21054791, and 21055959) shared a similarity of 99% with only 89% of the plasmid covered, when compared with publicly available IncX3 plasmids containing *bla*_OXA-181_ using Blast (Figure 3).

**Figure 4:**
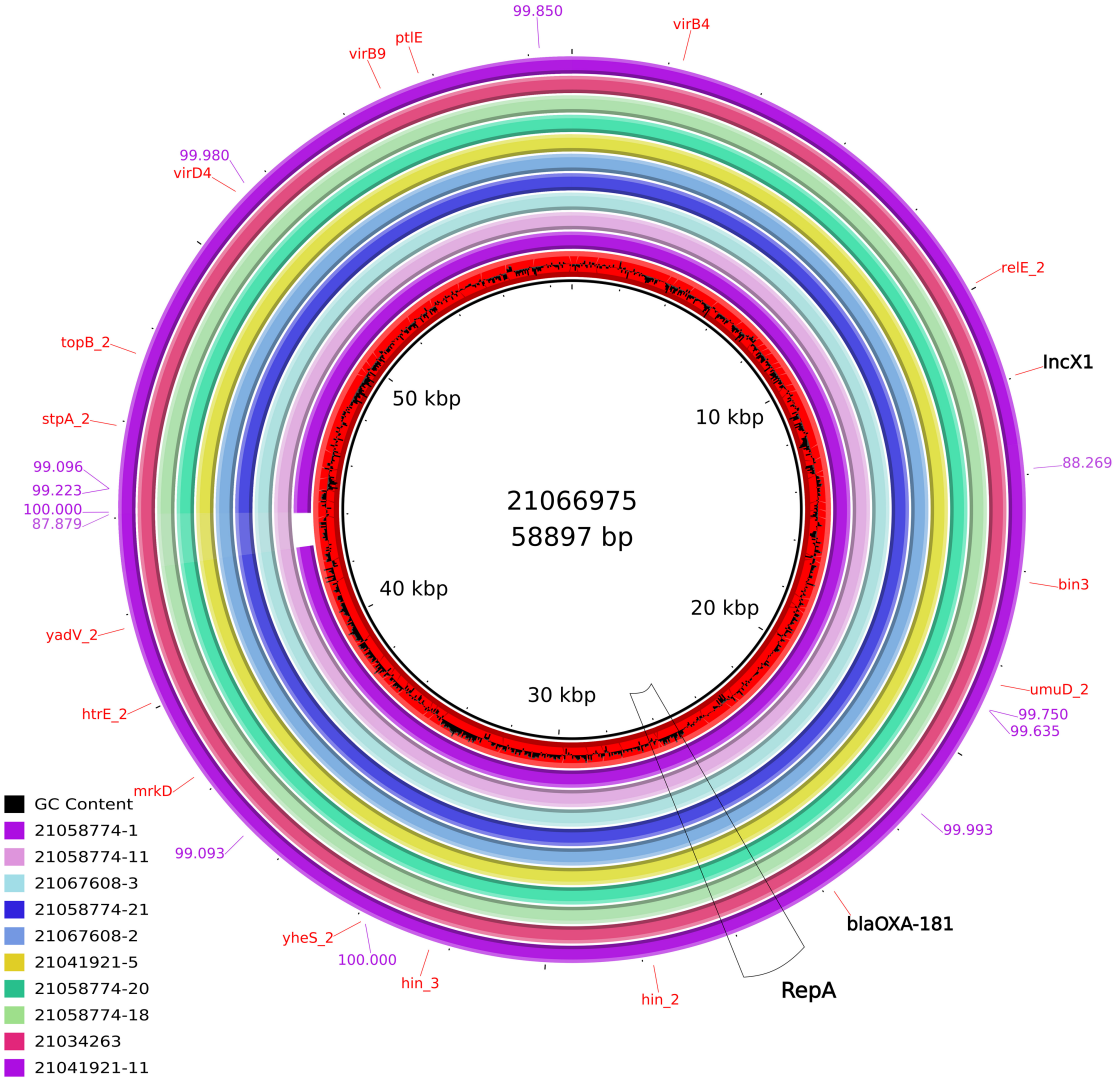
Comparison of the IncX1 plasmids from *E.coli* obtained in this study containing *bla*_OXA-181_. From the inner circle, the plasmids represented were: ID21066975 used as reference (accession number ERS12413440, *Escherichia coli,* pig, caecal content at slaughterhouse) ID21058774-1 (Breeding pig), ID21058774-11 (Breeding pig), ID21067608-3 (Human), ID21058774-21 (Breeding pig), ID21067608-2 (Human), ID21041921-5 (Fattening pig), ID21058774-20 (Breeding pig), ID21058774-18 (Breeding pig), ID21034263-1 (pig, caecal content at slaughterhouse), and ID21041921-11 (Fattening pig). The bluish values indicate the % of identity of one IncX1 complete sequence (ID 21041921–11) compared with the reference sequence ID 21066975.

At least, two IncX3 plasmids (IDs 21019054, 21055959) resolved in this study harbored a gene encoding a membrane transport protein (*yheS*) together with a DNA invertase. These genes were located in the “extra region” which was not present in the plasmids previously published. As for all the 12 IncX1 plasmids resolved in the present work, their size range was 57,694–58,897 bp and the largest one was used as reference (ID 21066975; pMOL6975). These plasmids were all almost identical between them with a 98-99% coverage and 99-100% sequence identity (Supplementary Table 1) and no similar IncX1 plasmids were found in public available databases. Our IncX1 plasmids contained mainly the genes involved in their maintenance, replication and conjugation (Figure 4). All harbored *bla*_OXA-181_ in a transposon (19869..28757), that also contained *rep*A and *ere*A, and flanked by *tn*3 and IS6. This IncX1 plasmid also presented a type II toxin/antitoxin system, with the toxin of the RelE/StbE family and its antitoxin RelB (9784..10306).

## Discussion

In the last years, *bla*_OXA-181_ has been detected not only in hospitals, but also in community settings, and occasionally also in animals (livestock, companion animals, and wildlife) and in the environment (Mairi et al., 2018). According to the EU-harmonized AMR monitoring program conducted in EU Member States using selective media for CRE, the occurrence of carbapenemase-producing *E. coli* and *Salmonella* spp. among livestock epidemiological Units in Europe has so far remained very sporadic. Indeed, only three *E.coli* isolates from Germany harboring *bla*_VIM-1_ (pig meat; Irrgang et al., 2019), *bla*_OXA-48_ (fattening pig; Irrgang et al., 2020a), *bla*_GES-5_ (fattening pig; Irrgang et al., 2020b), and one presumptive CR *E. coli* isolate from Romania, were detected in 2018 and 2019 [European Food Safety Authority (EFSA) and European Center for Disease Prevention and Control (ECDC), 2021]. In previous years (2015–2017), very similar results were also obtained, with only three *E. coli* from broilers and broiler meat in Romania confirmed as *bla*_OXA-162_ carriers [European Food Safety Authority (EFSA) and European Center for Disease Prevention and Control (ECDC), 2019; Bortolaia et al., 2021]. Prior to the findings described in this study, voluntary monitoring of CRE isolates conducted in Italy since 2014 revealed for the first time in 2019 the presence of only one *bla*_NDM-4_ positive *E.coli* from an Italian sample taken at slaughter (Diaconu et al., 2020). Conversely, thanks to the AMR monitoring activities conducted in 2021, we have witnessed for the first time the spread of OXA-181 producing isolates spreading in holdings of fattening pigs (estimated around 7%) and veal calves (around 2%), farmed in four contiguous Italian regions and provinces. This has been most likely facilitated by the intensive trade patterns occurring throughout the national territory, which is typical of these animal productions also across the EU [European Food Safety Authority (EFSA), 2010]. In our study, MLST analysis revealed a dominant ST (ST5229) in the pig and veal calf holdings in three out of the five Italian regions involved. Noteworthy, we detected the same ST also during the tracing back activities in the fattening holdings of origin, in different units of the same breeding holding, in the epidemiologically related dairy cattle holding and also in one of the two positive workers at the breeding holding (Table 3). To the best of our knowledge, *E. coli* ST5229 isolates have never been reported to harbor *bla*_OXA-48_-(like) genes. This ST has been previously described in *E.coli* harboring multiple AMR genes (e.g., *mcr* and ESBL genes) isolated from pig samples in different Asian countries (Li et al., 2021; Truong et al., 2021). Conversely, we observed that the same plasmids of three different Inc. groups carrying the *bla*_OXA-181_ gene in the same transposon structure were harbored by different STs. This feature is indicative of the mobilization of *bla*_OXA-181_ region and its likely horizontal transfer among different plasmid types and hosts. Accordingly, the massive and rapid dissemination of *bla*_OXA-181_ in the studied holdings could be the result of the combined effect of clonal spread and horizontal gene transfer.

Overall, clonal dissemination in humans has been previously reported to play a minor role in the spread of OXA-48-like carbapenemases, although certain high-risk clones of *Klebsiella pneumoniae* and *Escherichia coli* have been associated with the global spread of OXA-48, OXA-181, OXA-232, and OXA-204 carbapenemases in humans (Pitout et al., 2019). Among *E. coli* isolates, ST410 has been described as the most common high-risk global clone associated with *bla*_OXA-181_ in human patients, usually carried by IncX3 plasmid types (Pitout et al., 2019). Similarly, in animals this clone was also detected in 24 hospitalized pets in Switzerland (Nigg et al., 2019) and from a dog in Portugal (Brilhante et al., 2020). In our study, OXA-181 producing *E. coli* belonging to ST410 were identified in two caecal samples coming from two fattening pig holdings located in regions B and C (IDs 21100090–1 and 21100098–1) and this represents the first report of this high-risk clone associated with *bla*_OXA-181_ in livestock. This finding is of concern, as this clone has been reported to promote not only the spread of *bla*_OXA-181_, but also the spread of different carbapenem-resistant genes as *bla*_NDM_, *bla*_KPC-2_, and CTX-M-type extended-spectrum β-lactamase genes in clinical settings of different countries (Qin et al., 2018).

We have detected *bla*_OXA-181_ in three different plasmid types (IncX1, IncX3, and IncF types), which in most cases did not carry other AMR genes. However, almost all isolates showed a MDR profile and harbored the corresponding AMR genes, including co-resistance to HPCIAs (fluoroquinolones, macrolides, and third and fourth generation cephalosporins). Hence, these findings are of particular concern as they underline the co-occurrence, within the same isolate, of different plasmid types harboring multiple AMR genes, including those encoding resistance to last-resort drugs that are spread across the animal-human interface.

Moreover, the co-occurrence of different plasmids could facilitate the mobilization of the transposon containing *bla*_OXA-181_ to other MDR carrying plasmids or the fusion of these plasmids into a mosaic plasmid (Zheng et al., 2013).

In our study, we have detected *bla*_OXA-181_ in a composite transposon, in different *E. coli* isolated from pig and veal calf holdings of four Italian regions. So far, the global spread of *bla*_OXA-181_ among Enterobacterales in humans has been reported to be mainly caused by the same insertion element IS*Ecp1* situated within the Tn2013 transposon located on various plasmid backbones (Pitout et al., 2019). Additionally, it should be noted that we detected *bla*_OXA-181_ also in the same IncX3 plasmid of a MDR, AmpC producing (CMY-34-type) *C. freundii* (Figure 3). This isolate was also beta-lactam, fluoroquinolone and phenicol resistant, and was detected in the same pig holding which tested positive for OXA-181 producing MDR *E. coli* during the survey at slaughter in 2021 (data not shown). This finding provides the observational evidence that an identical OXA-181-positive plasmid (100% coverage and identity with the plasmid of ID 21019054) was shared between two Enterobacterales species isolated from the same porcine fecal sample. Moreover, in this study we have demonstrated that the same transposon containing *bla*_OXA-181_, could be found in plasmids belonging to at least three different Inc. groups, confirming that this gene may be itself mobilizable through transposable elements and could be introduced in different plasmid scaffolds.

This mobile structure was found to be identical to those previously detected in other IncX3 plasmids from *E. coli* isolated from human and occasional animal sources in different countries worldwide, and also in different Enterobacterales species as *Klebsiella pneumoniae* (Figure 3). In addition, the genetic environment of the *bla*_OXA-181_ gene observed in our plasmids was identical in approximately 80% of the sequence.

As for the IncX1 plasmids, to our knowledge, similar plasmids carrying *bla*_OXA-181_ have never been described before in Enterobacterales, as our IncX1 plasmids shared only an approximately 50% of identity with publicly available IncX1 sequences. In the isolates under study, this IncX1 plasmid is almost the same (99-100% identity), including the IncX1 plasmid found in OXA-181 producing *E. coli* from humans. In addition, IncX1 seems to be more stable than IncX3 probably because of the presence of the RelE/StbE toxin family and its antitoxin RelB in IncX1, as previously reported (Jensen and Gerdes, 1995). Overall, our study highlights that these plasmids can be efficiently harbored by *E. coli* ST/lineages frequently detected in fecal samples from pigs, calves and humans, and it is likely they are part of the “normal” intestinal microbiota of healthy food-producing animals, including those sent for slaughter. These findings are of public health importance due to the “opportunistic” nature and MDR attitude of Enterobacterales, which are a major threat in healthcare-related infections.

Although OXA-181 is a stronger carbapenemase than OXA-48, imipenem and meropenem MICs values are usually lower than for *K. pneumoniae* carbapenemase (KPC)-producing or Metallo-Beta-Lactamase (MBL)-positive isolates, and often remain apparently “susceptible” to some carbapenems according to current clinical EUCAST and CLSI breakpoints. However, the phenotypes of the OXA-181 producing isolates under study were unequivocally resistant to ertapenem (and temocillin), while only 13/25 (52%) tested microbiologically resistant to meropenem and 5/25 (20%) microbiologically and clinically resistant to imipenem (Table 1). Indeed, detection of carbapenemases of the OXA-48 family in clinical settings may be challenging, as previously reviewed (Kidd et al., 2020), because they usually show a modest hydrolytic activity against carbapenems that could also vary in relation to the different OXA-48-like enzymes. Moreover, clinical practice demonstrates how the *in vitro* susceptibility against carbapenems may not predict the success for the treatment of OXA-48-like producing Enterobacterales. In this regard, OXA-48-like producing Enterobacterales are most likely underreported, as they could be hard to identify without targeted molecular analysis. Other factors, including bacterial species, enzyme expression, membrane permeability, and any other resistance mechanisms, may contribute to the resistance phenotype (Kidd et al., 2020). Overall, these aspects are of particular concern, not only for the clinical relevance of Enterobacterales encoding for OXA-181 and in general for OXA-48-like carbapenemases, in human medicine, where high case-fatality rates have been observed in healthcare-associated infections (Cuzon et al., 2011; Navarro-San Francisco et al., 2013; Rodríguez et al., 2021), but also in the context of possible transmission events and spread (of isolates and MGEs) that can occur between animals and humans.

In conclusion, carbapenem-resistant Enterobacterales are still uncommon in animals worldwide and so far have been considered very sporadic in the EU. However, the findings of our population and genomic studies underline how their continuous and specific monitoring in the EU in food-producing animals and along the food chain is of a great relevance, despite carbapenems are among beta-lactams that have never been authorized for veterinary use. Indeed, the extensive use of other antimicrobials, as well as the continuous oral usage of aminopenicillins, besides selecting for extended-spectrum cephalosporin resistance (Cavaco et al., 2008), may be of selective advantage also for the spread of carbapenemases in animal primary productions, following their introduction from human or human-related sources. These findings in the animal productions sector should further increase the awareness of this hidden threat for human health. In our study, possible transmission pathways among farms, between animals and in-contact humans were investigated, and point to a human source as the most likely cause for the introduction of the OXA-181 carrying plasmid (IncX1 type) in the breeding holding where initial tracing-back activities were conducted. Whatever the initial source, we have provided evidence that these CRE have been amplified within the intensive animal production systems, especially in pigs. The spread of CRE in food-producing animals is of particular concern in a “One Health” perspective, since it may soon represent an important and additional source of CRE exposure for humans along the food chain. In this regard, specific integrated policies for risk mitigation are needed, in order to reduce the burden of MDR carbapenemase-producing bacteria in humans and minimize the impact of any possible animal and environmental reservoirs.

## Data availability statement

Illumina raw reads of the 36 isolates and ONT raw reads from 16 selected isolates analyzed in this study, were submitted to the European Nucleotide Archive (http://www.ebi.ac.uk/ena) under the study accession number PRJEB54630. The single sample accession numbers of each isolate are reported in Table 2 and 3.

## Ethics statement

Human fecal samples from workers and owners were voluntary made available through the Local Health Competent Authorities, after giving informed consent to participate in the study. This work does not contain experimentation with animals. As reported in the main text, the studied samples were collected from (1) caecal samples, taken by the Competent Authorities (Veterinary Services) after the slaughtering (for food) in the frame of the harmonized European Monitoring for Antimicrobial resistance (EU Decision 2013/652 and 2020/1729) or (2) fresh fecal samples taken by the Competent Authorities (Veterinary Services) on the floor of the boxes (of the holdings) where the animals were kept.

## Author contributions

AF, VC, PA, and AB conceived and designed the experiments. FG and LC performed tracing-back activities. AI, PD, LS, ED, and RA performed the experiments. FB and ELD managed the data. ELD, PA, VC, AF, and AB analyzed the data. VC, PA, ELD, AF, and AB wrote the paper. All authors contributed to the article and approved the submitted version.

## Funding

This work was funded partly by the Italian Ministry of Health (Ricerca Corrente, grant N° IZSLT - PRC202002). The genomic work on the use of the combined approach of short and long-read sequencing to resolve the OXA-181-carrying plasmids was conducted in the framework of the Full Force project, supported by the European Union’s Horizon 2020 Research and Innovation programme under grant agreement No 773830: One Health European Joint Programme.

## Conflict of interest

The authors declare that the research was conducted in the absence of any commercial or financial relationships that could be construed as a potential conflict of interest.

## Publisher’s note

All claims expressed in this article are solely those of the authors and do not necessarily represent those of their affiliated organizations, or those of the publisher, the editors and the reviewers. Any product that may be evaluated in this article, or claim that may be made by its manufacturer, is not guaranteed or endorsed by the publisher.

## Footnotes

^1^ https://eur-lex.europa.eu/legal-content/EN/TXT/PDF/?uri=CELEX:32013D0652&from=EN

^2^ https://eur-lex.europa.eu/legal-content/EN/TXT/PDF/?uri=CELEX:32020D1729&from=EN

^3^ https://efsa.onlinelibrary.wiley.com/doi/epdf/10.2903/j.efsa.2020.6364

^4^ https://www.eurl-ar.eu/CustomerData/Files/Folders/21-protocols/530_esbl-ampc-cpeprotocol-version-caecal-v7-09-12-19.pdf

^5^ www.eucast.org

^6^ https://www.bioinformatics.babraham.ac.uk/projects/fastqc/

^7^ https://github.com/tseemann/mlst

^8^ https://github.com/tseemann/abricate

^9^ https://cge.cbs.dtu.dk/services/ResFinder/

^10^ https://cge.cbs.dtu.dk/services/PlasmidFinder/

^11^ https://cge.cbs.dtu.dk/services/SerotypeFinder/

^12^ https://cge.cbs.dtu.dk/services/VirulenceFinder/

^13^ http://www.mgc.ac.cn/VFs/

^14^ https://www.r-project.org
